# *N*,*N*′-Dicyclo­hexyl-*N*′′,*N*′′-dimethyl­phospho­ric triamide

**DOI:** 10.1107/S160053681100287X

**Published:** 2011-01-26

**Authors:** Fahimeh Sabbaghi, Mehrdad Pourayoubi, Fatemeh Karimi Ahmadabad, Zahra Azarkamanzad, Ali Asghar Ebrahimi Valmoozi

**Affiliations:** a Department of Chemistry, Islamic Azad University–Zanjan Branch, PO Box 49195-467, Zanjan, Iran; b Department of Chemistry, Ferdowsi University of Mashhad, Mashhad 91779, Iran; c Department of Chemistry, Tarbiat Modares University, Tehran, Iran

## Abstract

In the title compound, C_14_H_30_N_3_OP, both cyclo­hexyl groups adopt chair conformations with the NH unit in an equatorial position. The P atom adopts a slightly distorted tetra­hedral environment. In the (CH_3_)_2_NP(O) unit, the O—P—N—C torsion angles, showing the orientations of the methyl groups with respect to the phosphoryl group, are −166.6 (3) and 34.6 (4)°. The O atom of the P=O group acts as a double hydrogen-bond acceptor and is involved in two different inter­molecular N—H⋯OP hydrogen bonds, building *R*_2_^2^(8) rings that are further linked into chains running parallel to the *b* axis.

## Related literature

For the structure of a phospho­ramidate with a [(CH_3_)_2_N]P(O) unit, see: Ghadimi *et al.* (2009 ▶). For bond distances in related structures, see: Sabbaghi *et al.* (2010 ▶). For hydrogen-bond motifs, see: Etter *et al.* (1990 ▶); Bernstein *et al.* (1995 ▶). For double hydrogen-bond acceptors, see: Steiner (2002 ▶).
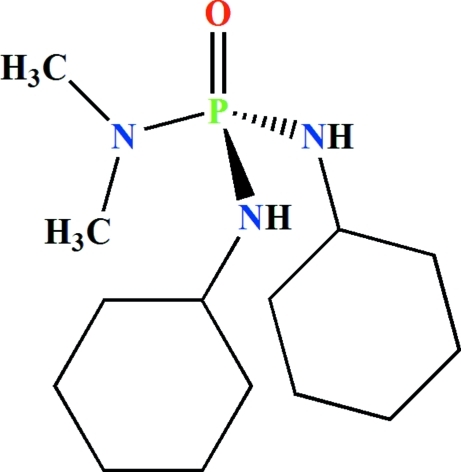


## Experimental

### 

#### Crystal data

C_14_H_30_N_3_OP
*M* *_r_* = 287.38
Monoclinic, 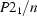

*a* = 11.742 (4) Å
*b* = 7.712 (3) Å
*c* = 18.366 (6) Å
β = 102.120 (7)°
*V* = 1626.0 (10) Å^3^
*Z* = 4
Mo *K*α radiation
μ = 0.17 mm^−1^
*T* = 120 K
0.23 × 0.19 × 0.13 mm

#### Data collection

Bruker SMART 1000 CCD area-detector diffractometer
Absorption correction: multi-scan (*SADABS*; Sheldrick, 1998 ▶) *T*_min_ = 0.932, *T*_max_ = 0.974
11336 measured reflections
3507 independent reflections
1873 reflections with *I* > 2σ(*I*)
*R*_int_ = 0.103

#### Refinement

*R*[*F* ^2^ > 2σ(*F* ^2^)] = 0.072
*wR*(*F* ^2^) = 0.199
*S* = 1.04
3507 reflections
174 parameters
H-atom parameters constrained
Δρ_max_ = 0.51 e Å^−3^
Δρ_min_ = −0.47 e Å^−3^

### 

Data collection: *SMART* (Bruker, 1998 ▶); cell refinement: *SAINT-Plus* (Bruker, 1998 ▶); data reduction: *SAINT-Plus*; program(s) used to solve structure: *SHELXTL* (Sheldrick, 2008 ▶); program(s) used to refine structure: *SHELXTL*; molecular graphics: *SHELXTL* and *Mercury* (Macrae *et al.*, 2008 ▶); software used to prepare material for publication: *SHELXTL*.

## Supplementary Material

Crystal structure: contains datablocks I, global. DOI: 10.1107/S160053681100287X/nc2217sup1.cif

Structure factors: contains datablocks I. DOI: 10.1107/S160053681100287X/nc2217Isup2.hkl

Additional supplementary materials:  crystallographic information; 3D view; checkCIF report

## Figures and Tables

**Table 1:** Hydrogen-bond geometry (Å, °)

*D*—H⋯*A*	*D*—H	H⋯*A*	*D*⋯*A*	*D*—H⋯*A*
N2—H2⋯O1^i^	0.90	2.16	3.017 (4)	160
N3—H3⋯O1^ii^	0.90	2.03	2.911 (4)	165

Symmetry codes: (i) 
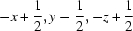
; (ii) 
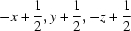
.

**Fig. 1.**
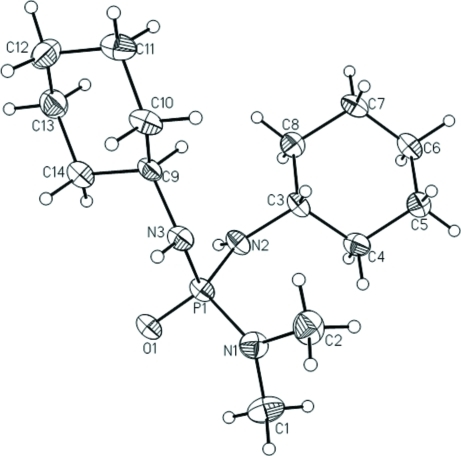
Molecular structure of title compound with labeling and displacement ellipsoids drawn at the 50% probability level.

**Fig. 2.**
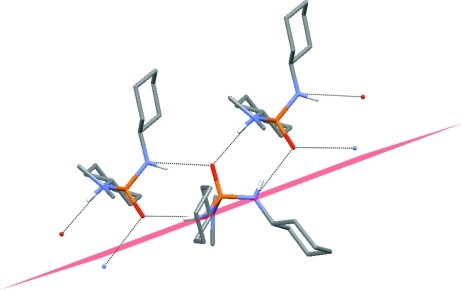
Part of the crystal structure of the title compound with hydrogen bonding shown as dotted lines (the C—H hydrogen atoms are omitted for clarity).

## supplementary crystallographic information

### Comment

The structure determination was performed as a part of a project on the synthesis of new phosphorus compounds having a [(CH_3_)_2_N]P(O) moiety (Ghadimi *et al.*, 2009).

In the crystal structure of the title compound the two cyclohexyl groups are in a chair conformation with the NH units in equatorial positions (Fig. 1). The P atom is in a slightly distorted tetrahedral environment with bond angles in the range of 100.87 (17)° [N3—P1— N1] to 118.64 (16)° [O1—P1—N3]. In the (CH_3_)_2_NP(O) moiety, the dihedral angles O—P—N—C are -166.6 (3)° and 34.6 (4)°. The P—N bond lengths are comparable to those in similar compounds like for example in P(O)[NHC(O)C_6_H_4_(4-NO_2_)][NHC_6_H_11_]_2_ (Sabbaghi *et al.*, 2010).

The molecules are linked by two intermolecular N—H···OP hydrogen bonds into chains in the direction of the *b* axis in which the O atom of the P═O group acts as a double H-acceptors (Steiner, 2002) (Fig. 2). From this arrangement *R*_2_^2^(8) rings are formed (Etter *et al.*, 1990; Bernstein *et al.*, 1995).

### Experimental

Synthesis of ((CH_3_)_2_N)P(O)Cl_2_ [(CH_3_)_2_NH_2_]Cl (15.00 g, 0.184 mol) and P(O)Cl_3_ (84.62 g, 0.552 mol) were refluxed for 8 h and afterwards the excess of P(O)Cl_3_ was removed in vacuum.

Synthesis of title compound To a solution of ((CH_3_)_2_N)P(O)Cl_2_ (0.60 g, 3.7 mmol) in chloroform (15 mL), a solution of cyclohexylamine (1.47 g, 14.8 mmol) in chloroform (10 mL) was added at 273 K. After 4 h stirring, the solvent was removed and product was washed with deionized water and recrystallized from chloroform/methanol (4:1 v/v) at room temperature.

### Refinement

The hydrogen atoms of NH groups were located by difference Fourier synthesis and normalized at standard value 0.90 Å, whereas the C-H H atoms were positioned with idealized geometry. All H atoms were refined isotropic with *U*_iso_(H) = 1.2*U*_eq_(C, N) (1.5 for methyl H atoms) using a riding model.

### Crystal data

**Table:**

C_14_H_30_N_3_OP	*F*(000) = 632
*M**_r_* = 287.38	*D*_x_ = 1.174 Mg m^−^^3^
Monoclinic, *P*2_1_/*n*	Mo *K*α radiation, λ = 0.71073 Å
Hall symbol: -P 2yn	Cell parameters from 1063 reflections
*a* = 11.742 (4) Å	θ = 2.3–26.9°
*b* = 7.712 (3) Å	µ = 0.17 mm^−^^1^
*c* = 18.366 (6) Å	*T* = 120 K
β = 102.120 (7)°	Prizm, colorless
*V* = 1626.0 (10) Å^3^	0.23 × 0.19 × 0.13 mm
*Z* = 4	

### Data collection

**Table:**

Bruker SMART 1000 CCD area-detector diffractometer	3507 independent reflections
Radiation source: normal-focus sealed tube	1873 reflections with *I* > 2σ(*I*)
graphite	*R*_int_ = 0.103
φ and ω scans	θ_max_ = 27.0°, θ_min_ = 1.9°
Absorption correction: multi-scan (*SADABS*; Sheldrick, 1998)	*h* = −15→14
*T*_min_ = 0.932, *T*_max_ = 0.974	*k* = −9→9
11336 measured reflections	*l* = −23→22

### Refinement

**Table:**

Refinement on *F*^2^	Primary atom site location: structure-invariant direct methods
Least-squares matrix: full	Secondary atom site location: difference Fourier map
*R*[*F*^2^ > 2σ(*F*^2^)] = 0.072	Hydrogen site location: mixed
*wR*(*F*^2^) = 0.199	H-atom parameters constrained
*S* = 1.04	*w* = 1/[σ^2^(*F*_o_^2^) + (0.062*P*)^2^ + 2.1669*P*] where *P* = (*F*_o_^2^ + 2*F*_c_^2^)/3
3507 reflections	(Δ/σ)_max_ < 0.001
174 parameters	Δρ_max_ = 0.51 e Å^−^^3^
0 restraints	Δρ_min_ = −0.47 e Å^−^^3^

### Special details

**Table:**

Geometry. All e.s.d.'s (except the e.s.d. in the dihedral angle between two l.s. planes) are estimated using the full covariance matrix. The cell e.s.d.'s are taken into account individually in the estimation of e.s.d.'s in distances, angles and torsion angles; correlations between e.s.d.'s in cell parameters are only used when they are defined by crystal symmetry. An approximate (isotropic) treatment of cell e.s.d.'s is used for estimating e.s.d.'s involving l.s. planes.
Refinement. Refinement of *F*^2^ against ALL reflections. The weighted *R*-factor *wR* and goodness of fit *S* are based on *F*^2^, conventional *R*-factors *R* are based on *F*, with *F* set to zero for negative *F*^2^. The threshold expression of *F*^2^ > σ(*F*^2^) is used only for calculating *R*-factors(gt) *etc*. and is not relevant to the choice of reflections for refinement. *R*-factors based on *F*^2^ are statistically about twice as large as those based on *F*, and *R*- factors based on ALL data will be even larger.

### Fractional atomic coordinates and isotropic or equivalent isotropic displacement parameters (Å^2^)

**Table:**

	*x*	*y*	*z*	*U*_iso_*/*U*_eq_	
P1	0.15685 (9)	0.10911 (13)	0.22176 (6)	0.0231 (3)	
O1	0.2842 (2)	0.0799 (3)	0.23095 (15)	0.0261 (6)	
N1	0.1077 (3)	0.2079 (4)	0.14150 (19)	0.0287 (8)	
N2	0.0944 (3)	−0.0770 (4)	0.23092 (18)	0.0250 (8)	
H2	0.1465	−0.1633	0.2435	0.030*	
N3	0.1111 (3)	0.2411 (4)	0.27929 (18)	0.0242 (8)	
H3	0.1359	0.3518	0.2819	0.029*	
C1	0.1595 (4)	0.1671 (6)	0.0782 (2)	0.0385 (11)	
H1A	0.1662	0.2733	0.0501	0.058*	
H1B	0.1101	0.0834	0.0458	0.058*	
H1C	0.2371	0.1170	0.0960	0.058*	
C2	−0.0065 (4)	0.2895 (6)	0.1205 (3)	0.0371 (11)	
H2A	0.0010	0.4007	0.0961	0.056*	
H2B	−0.0381	0.3090	0.1652	0.056*	
H2C	−0.0591	0.2135	0.0861	0.056*	
C3	−0.0309 (3)	−0.1127 (5)	0.2152 (2)	0.0229 (8)	
H3A	−0.0725	−0.0033	0.2230	0.027*	
C4	−0.0756 (3)	−0.1728 (5)	0.1348 (2)	0.0281 (9)	
H4A	−0.0616	−0.0807	0.1002	0.034*	
H4B	−0.0320	−0.2774	0.1252	0.034*	
C5	−0.2050 (4)	−0.2142 (6)	0.1202 (2)	0.0302 (10)	
H5A	−0.2312	−0.2551	0.0682	0.036*	
H5B	−0.2492	−0.1079	0.1264	0.036*	
C6	−0.2297 (4)	−0.3535 (5)	0.1737 (2)	0.0299 (10)	
H6A	−0.3145	−0.3766	0.1642	0.036*	
H6B	−0.1899	−0.4624	0.1651	0.036*	
C7	−0.1876 (3)	−0.2957 (5)	0.2534 (2)	0.0279 (9)	
H7A	−0.2012	−0.3897	0.2873	0.033*	
H7B	−0.2329	−0.1931	0.2631	0.033*	
C8	−0.0574 (4)	−0.2497 (5)	0.2699 (2)	0.0272 (9)	
H8A	−0.0113	−0.3555	0.2661	0.033*	
H8B	−0.0342	−0.2049	0.3214	0.033*	
C9	0.1053 (3)	0.1887 (5)	0.3554 (2)	0.0229 (9)	
H9A	0.0596	0.0784	0.3517	0.027*	
C10	0.0390 (4)	0.3243 (5)	0.3902 (2)	0.0295 (10)	
H10A	−0.0386	0.3432	0.3576	0.035*	
H10B	0.0819	0.4356	0.3947	0.035*	
C11	0.0243 (4)	0.2654 (6)	0.4670 (3)	0.0362 (11)	
H11A	−0.0234	0.1586	0.4621	0.043*	
H11B	−0.0168	0.3563	0.4895	0.043*	
C12	0.1444 (4)	0.2298 (6)	0.5181 (2)	0.0385 (11)	
H12A	0.1894	0.3391	0.5268	0.046*	
H12B	0.1334	0.1863	0.5669	0.046*	
C13	0.2116 (4)	0.0969 (6)	0.4830 (2)	0.0362 (10)	
H13A	0.1707	−0.0160	0.4797	0.043*	
H13B	0.2900	0.0814	0.5150	0.043*	
C14	0.2238 (3)	0.1534 (6)	0.4049 (2)	0.0298 (10)	
H14A	0.2720	0.2596	0.4086	0.036*	
H14B	0.2637	0.0611	0.3822	0.036*	

### Atomic displacement parameters (Å^2^)

**Table:**

	*U*^11^	*U*^22^	*U*^33^	*U*^12^	*U*^13^	*U*^23^
P1	0.0193 (5)	0.0217 (5)	0.0295 (6)	−0.0005 (4)	0.0079 (4)	−0.0001 (4)
O1	0.0198 (14)	0.0238 (15)	0.0363 (16)	−0.0024 (11)	0.0096 (12)	−0.0017 (12)
N1	0.028 (2)	0.0280 (19)	0.031 (2)	0.0039 (15)	0.0084 (15)	0.0020 (15)
N2	0.0175 (17)	0.0233 (18)	0.0352 (19)	0.0007 (13)	0.0077 (14)	0.0006 (14)
N3	0.0225 (18)	0.0228 (18)	0.0289 (19)	−0.0022 (13)	0.0087 (14)	−0.0016 (14)
C1	0.055 (3)	0.030 (2)	0.034 (3)	0.004 (2)	0.019 (2)	0.0054 (19)
C2	0.033 (3)	0.034 (2)	0.043 (3)	0.006 (2)	0.003 (2)	0.007 (2)
C3	0.0155 (19)	0.0207 (19)	0.033 (2)	−0.0009 (16)	0.0064 (16)	−0.0002 (17)
C4	0.029 (2)	0.027 (2)	0.031 (2)	−0.0042 (18)	0.0119 (18)	−0.0033 (18)
C5	0.026 (2)	0.036 (2)	0.029 (2)	−0.0036 (18)	0.0054 (18)	−0.0019 (18)
C6	0.021 (2)	0.029 (2)	0.041 (3)	−0.0014 (17)	0.0086 (18)	−0.0020 (19)
C7	0.024 (2)	0.025 (2)	0.038 (2)	−0.0022 (17)	0.0133 (18)	0.0024 (18)
C8	0.026 (2)	0.025 (2)	0.031 (2)	0.0017 (17)	0.0058 (17)	0.0008 (17)
C9	0.019 (2)	0.023 (2)	0.029 (2)	−0.0053 (16)	0.0085 (16)	−0.0014 (17)
C10	0.028 (2)	0.026 (2)	0.039 (2)	−0.0008 (18)	0.0164 (19)	0.0023 (18)
C11	0.038 (3)	0.036 (3)	0.041 (3)	0.000 (2)	0.020 (2)	−0.001 (2)
C12	0.046 (3)	0.045 (3)	0.024 (2)	−0.007 (2)	0.006 (2)	−0.002 (2)
C13	0.031 (2)	0.041 (3)	0.036 (2)	−0.005 (2)	0.0048 (19)	0.006 (2)
C14	0.024 (2)	0.031 (2)	0.035 (2)	−0.0026 (17)	0.0082 (18)	0.0004 (18)

### Geometric parameters (Å, °)

**Table:**

P1—O1	1.486 (3)	C6—C7	1.511 (6)
P1—N2	1.636 (3)	C6—H6A	0.9900
P1—N3	1.637 (3)	C6—H6B	0.9900
P1—N1	1.652 (4)	C7—C8	1.536 (6)
N1—C2	1.457 (5)	C7—H7A	0.9900
N1—C1	1.456 (5)	C7—H7B	0.9900
N2—C3	1.465 (5)	C8—H8A	0.9900
N2—H2	0.9000	C8—H8B	0.9900
N3—C9	1.471 (5)	C9—C14	1.518 (5)
N3—H3	0.9000	C9—C10	1.522 (5)
C1—H1A	0.9800	C9—H9A	1.0000
C1—H1B	0.9800	C10—C11	1.526 (6)
C1—H1C	0.9800	C10—H10A	0.9900
C2—H2A	0.9800	C10—H10B	0.9900
C2—H2B	0.9800	C11—C12	1.545 (6)
C2—H2C	0.9800	C11—H11A	0.9900
C3—C4	1.532 (5)	C11—H11B	0.9900
C3—C8	1.534 (5)	C12—C13	1.518 (6)
C3—H3A	1.0000	C12—H12A	0.9900
C4—C5	1.521 (6)	C12—H12B	0.9900
C4—H4A	0.9900	C13—C14	1.535 (6)
C4—H4B	0.9900	C13—H13A	0.9900
C5—C6	1.524 (6)	C13—H13B	0.9900
C5—H5A	0.9900	C14—H14A	0.9900
C5—H5B	0.9900	C14—H14B	0.9900
			
O1—P1—N2	108.49 (16)	H6A—C6—H6B	108.1
O1—P1—N3	118.64 (16)	C6—C7—C8	111.7 (3)
N2—P1—N3	105.33 (17)	C6—C7—H7A	109.3
O1—P1—N1	109.04 (17)	C8—C7—H7A	109.3
N2—P1—N1	114.59 (17)	C6—C7—H7B	109.3
N3—P1—N1	100.87 (17)	C8—C7—H7B	109.3
C2—N1—C1	113.4 (3)	H7A—C7—H7B	108.0
C2—N1—P1	124.4 (3)	C7—C8—C3	111.1 (3)
C1—N1—P1	119.1 (3)	C7—C8—H8A	109.4
C3—N2—P1	126.7 (3)	C3—C8—H8A	109.4
C3—N2—H2	120.8	C7—C8—H8B	109.4
P1—N2—H2	112.3	C3—C8—H8B	109.4
C9—N3—P1	122.0 (3)	H8A—C8—H8B	108.0
C9—N3—H3	106.8	N3—C9—C14	113.4 (3)
P1—N3—H3	118.5	N3—C9—C10	109.9 (3)
N1—C1—H1A	109.5	C14—C9—C10	111.0 (3)
N1—C1—H1B	109.5	N3—C9—H9A	107.4
H1A—C1—H1B	109.5	C14—C9—H9A	107.4
N1—C1—H1C	109.5	C10—C9—H9A	107.4
H1A—C1—H1C	109.5	C9—C10—C11	110.5 (3)
H1B—C1—H1C	109.5	C9—C10—H10A	109.6
N1—C2—H2A	109.5	C11—C10—H10A	109.6
N1—C2—H2B	109.5	C9—C10—H10B	109.6
H2A—C2—H2B	109.5	C11—C10—H10B	109.6
N1—C2—H2C	109.5	H10A—C10—H10B	108.1
H2A—C2—H2C	109.5	C10—C11—C12	110.5 (3)
H2B—C2—H2C	109.5	C10—C11—H11A	109.6
N2—C3—C4	111.9 (3)	C12—C11—H11A	109.6
N2—C3—C8	109.5 (3)	C10—C11—H11B	109.6
C4—C3—C8	110.3 (3)	C12—C11—H11B	109.6
N2—C3—H3A	108.3	H11A—C11—H11B	108.1
C4—C3—H3A	108.3	C13—C12—C11	110.5 (4)
C8—C3—H3A	108.3	C13—C12—H12A	109.5
C5—C4—C3	111.2 (3)	C11—C12—H12A	109.5
C5—C4—H4A	109.4	C13—C12—H12B	109.5
C3—C4—H4A	109.4	C11—C12—H12B	109.5
C5—C4—H4B	109.4	H12A—C12—H12B	108.1
C3—C4—H4B	109.4	C12—C13—C14	111.4 (4)
H4A—C4—H4B	108.0	C12—C13—H13A	109.3
C4—C5—C6	110.6 (3)	C14—C13—H13A	109.3
C4—C5—H5A	109.5	C12—C13—H13B	109.3
C6—C5—H5A	109.5	C14—C13—H13B	109.3
C4—C5—H5B	109.5	H13A—C13—H13B	108.0
C6—C5—H5B	109.5	C9—C14—C13	110.9 (3)
H5A—C5—H5B	108.1	C9—C14—H14A	109.5
C7—C6—C5	110.4 (3)	C13—C14—H14A	109.5
C7—C6—H6A	109.6	C9—C14—H14B	109.5
C5—C6—H6A	109.6	C13—C14—H14B	109.5
C7—C6—H6B	109.6	H14A—C14—H14B	108.1
C5—C6—H6B	109.6		
			
O1—P1—N1—C2	−166.6 (3)	C3—C4—C5—C6	−58.0 (4)
N2—P1—N1—C2	71.6 (4)	C4—C5—C6—C7	57.8 (4)
N3—P1—N1—C2	−40.9 (4)	C5—C6—C7—C8	−56.5 (4)
O1—P1—N1—C1	34.6 (4)	C6—C7—C8—C3	55.2 (4)
N2—P1—N1—C1	−87.2 (3)	N2—C3—C8—C7	−177.9 (3)
N3—P1—N1—C1	160.2 (3)	C4—C3—C8—C7	−54.3 (4)
O1—P1—N2—C3	−170.3 (3)	P1—N3—C9—C14	65.7 (4)
N3—P1—N2—C3	61.7 (3)	P1—N3—C9—C10	−169.4 (3)
N1—P1—N2—C3	−48.2 (4)	N3—C9—C10—C11	175.8 (3)
O1—P1—N3—C9	−77.8 (3)	C14—C9—C10—C11	−57.9 (4)
N2—P1—N3—C9	43.8 (3)	C9—C10—C11—C12	57.5 (5)
N1—P1—N3—C9	163.3 (3)	C10—C11—C12—C13	−56.5 (5)
P1—N2—C3—C4	91.1 (4)	C11—C12—C13—C14	55.4 (5)
P1—N2—C3—C8	−146.3 (3)	N3—C9—C14—C13	−179.2 (3)
N2—C3—C4—C5	178.3 (3)	C10—C9—C14—C13	56.6 (4)
C8—C3—C4—C5	56.1 (4)	C12—C13—C14—C9	−55.7 (5)

### Hydrogen-bond geometry (Å, °)

**Table:**

*D*—H···*A*	*D*—H	H···*A*	*D*···*A*	*D*—H···*A*
N2—H2···O1^i^	0.90	2.16	3.017 (4)	160
N3—H3···O1^ii^	0.90	2.03	2.911 (4)	165

Symmetry codes: (i) −*x*+1/2, *y*−1/2, −*z*+1/2; (ii) −*x*+1/2, *y*+1/2, −*z*+1/2.
